# Coagulopathy: Another side effect of coronavirus infection

**DOI:** 10.34172/jcvtr.2020.59

**Published:** 2020-12-23

**Authors:** Fatemeh Sadoughi, Parisa Maleki Dana, Jamal Hallajzadeh, Zatollah Asemi, Mohammad Ali Mansournia, Bahman Yousefi

**Affiliations:** ^1^Research Center for Biochemistry and Nutrition in Metabolic Diseases, Kashan University of Medical Sciences, Kashan, Iran; ^2^Department of Biochemistry and Nutrition, Research Center for Evidence-Based Health Management, Maragheh University of Medical Sciences, Maragheh, Iran; ^3^Department of Epidemiology and Biostatistics, School of Public Health, Tehran University of Medical Sciences, Tehran, Iran; ^4^Stem Cell Research Center, Tabriz University of Medical Sciences, Tabriz, Iran

**Keywords:** SARS, COVID-19, CoV, Heparin, DIC, PIC, VTE

## Abstract

Recently, coronavirus disease 2019 (COVID-19) has been considered as a major health problem around the globe. This severe acute respiratory syndrome has a bunch of features, such as high transmission rate, which are adding to its importance. Overcoming this disease relies on a complete understanding of the viral structure, receptors, at-risk cells or tissues, and pathogenesis. Currently, researches have shown that besides the lack of a proper anti-viral therapeutic method, complications provided by this virus are also standing in the way of decreasing its mortality rate. One of these complications is believed to be a hematologic manifestation. Commonly, three kinds of coagulopathies are detected in COVID-19 patients: disseminated intravascular coagulation (DIC), pulmonary embolism (PE), and deep vein thrombosis (DVT). In this paper, we have reviewed the relation between these conditions and coronavirus-related diseases pathogenesis, severity, and mortality rate.

## Introduction

During the past two decades, members of coronaviridae family have caused severe global economic and health problems on a large scale. Three species of coronaviruses called SARS-CoV-1, Middle East Respiratory Syndrome (MERS), and SARS-CoV-2 are responsible for three outbreaks which occurred in 2002, 2012, and 2019, respectively.^1-3^ According to statistics, MERS, SARS, and COVID-19 or 2019-nCoV have the mortality rate of 35%,^1^ 11%,^3^ and 4.3% to 14.6%^2^, respectively. However, the importance of these diseases is not only related to the respiratory disturbance, the number of deaths, or the high transmission rate of these viruses.^2^ The fact that other organs of our body such as brain, heart, and kidneys are also prone to be affected by coronaviruses is making the process of treatment more complicated and the disease prognosis poorer. In this regard, cardiovascular, metabolic, renal, and nervous system disorders have been observed in patients infected with coronaviruses.^4-9^ Currently, the association of these viruses has been reported with a life-threatening condition: coagulopathy.^10^

The name of coagulopathy is attributed to a condition which the balance between pro-coagulant and anti-coagulant factors is impaired and therefore, blood loses its ability of clot formation or clot degradation. In other words, coagulopathy can also be defined as whether excessive bleeding or clotting.^11^ Noteworthy, in some conditions such as disseminated intravascular coagulation (DIC), in contrast to thrombocytopenia, fibrin deposition can also occur.^12^ Some factors such as trauma, sepsis, consumption of some drugs, and genetic diseases are able to induce this condition by altering the amounts of plasma proteins involved in the process of coagulation.^13-17^ Regardless of the etiology of this condition, internal hemorrhage, organ dysfunction, and thrombotic obstruction followed by excessive bleeding are the reasons why this condition can be lethal.^12,13^

Overall, the orientation of a great amount of evidence has changed towards the diseases caused by coronaviruses not only for preventing more deaths to happen but also for enhancing the life quality of survivors which are the majority of the infected population. To that end, we have conducted a review on coagulation disorders which are one of the complications provided by coronaviruses. This paper might be a great help for facilitating the treatment process, decreasing the mortality rate, and increasing the life quality of CoV-related diseases.

## Discussion

### Coronaviruses and pathogenesis: mechanisms

Generally, coronaviruses have the capability of infecting a number of animal species but the ones we are discussing only affect mammals.^18^ Although, before investigating the mechanisms of coronavirus pathogenesis, it is necessary to have a complete understanding of their structure and components. In general, all coronaviruses are composed of a positive RNA and two other ingredients that are made up of proteins: capsid and envelope.^19^ Attachment, entrance, replication, and overall every step of the viral pathogenesis relies on proteins.^20^ In the coronavirus structure, five different proteins exist: nucleocapsid, membrane, envelope, spike, and envelope-associated hemagglutinin-esterase proteins.^18,20^ Among all these proteins, spike proteins have the most important roles in receptor binding, membrane fusion, entry, and immune response induction.^18^

The genus of coronavirus contains four generas: alpha, beta, gamma, and delta. These subgroups are different in some properties, such as the kind of receptor they recognize.^20^ However, members of a common subgroup might also recognize distinct receptors.^18^ For instance, SARS-CoV-1, SARS-CoV-2, and MERS-CoV are all attributed to the beta subgroup of coronaviruses. However, the first two viruses recognize angiotensin-converting enzyme 2 (ACE2) which is a zinc peptidase but the last one binds to dipeptidyl peptidase 4 (DPP4).^21-23^ These receptors have a significant role in our understanding of coronavirus pathogenesis. ACE2 is a homolog of angiotensin-converting enzyme and is able to generate angiotensin 1-9 from angiotensin I and angiotensin 1-7 from angiotensin II.^24^ These functions are allowing ACE2 to have an impact on the renin-angiotensin system; thereby, affecting the cardiac and renal function.^24,25^ However, the expression of this enzyme is not limited to heart and kidneys and it also can be found in lungs and colon.^25^ Recently, researches have shown that post-CoV-infection lung failure and cardiovascular diseases (CVDs) are happening due to the down-regulation of ACE2.^26,27^

### SARS-CoV-2 and coagulopathy

The need for understanding all the aspects of coronavirus influence on diverse cells and organs of our body in addition to the complex treatment process for COVID-19 patients has oriented investigation towards revealing the effect of coronaviruses on other systems than the respiratory system. In this regard, the coagulation process has been detected to be disturbed by COVID-19.

### Mechanisms

Despite the incompleteness of our knowledge on the exact mechanisms by which COVID-19 is causing coagulopathy, evidence suggest that ACE2, intensified inflammation, and complement activation are three major basics of this event.

### Inflammation

It seems that intensifeied inflammation is the reason of the activation of coagulation cascade in COVID-19 patients. It is assumed primary origin of coagulopathy is in the pulmonary system and is caused by diffuse bilateral inflammation.^28^ This condition which is different from DIC is called pulmonary intravascular coagulopathy (PIC).^29^

After infection, a condition occurs in the patient’s body which is caused by the over-production of some proinflammatory cytokines and is called cytokine storm or macrophage activation syndrome (MAS).^29,30^ It is approved that cytokine storm plays a pivotal role in causing damages to diverse organs and systems of our body after being infected by SARS-CoV-2.^30^ Micro-thrombosis and haemorrhage are two of these damages which are locally occurring in pulmonary vessels.^29^ Thus, MAS is the major basic of causing PIC in COVID-19 patients.

Moreover, DIC is also observed in these patients which has lower dependency to MAS than PIC is attributed to the systemic activation of macrophages.^29^

### ACE2

Considering the expression pattern of ACE2 on endothelial cells of different organs, SARS-CoV-2 might also cause coagulopathy by its receptor.^31^ It is reported that after the binding of SARS-CoV-2 to ACE2 endothelitis can be observed heart, kidneys, and liver along with lungs.^31^ Triggered hypercoagulable state after endothelitis occurs due to excesive thrombin production and fibrinolysis shutdown.

However, still, it is not completely clear that endothelium dysfunction happens due to whether ACE2 or inflammation and therefore, further investigations are needed for clarifying the exact role of ACE2 in coagulopathy.

### Complement activation

There is only one study investigating the role of complement activation in coagulopathy pathogenesis and this means that there is a lot of room for more examinations. In this study, microvascular injury was detected in 5 COVID-19 patients which had “deposits of terminal complement components C5b-9 (membrane attack complex), C4d, and mannose binding lectin (MBL)-associated serine protease (MASP)2” in their pulmonary tissue.^32^

After all, the association of complement with coagulopathy in COVID patients is not definite.

### Prevalence and varieties of coagulation disorders

In general, it seems that there is a relation between pneumonias and coaguloathies regardless to the reason causing pneumonia.^33^ Interestingly, comparing coagulation states in COVID and non-COVID patients has shown that SARS-CoV-2 is able to increase the number of serum platelets to a higher level than other pathogens and thereby, twofold the risk of coagulopathy.^33^

Till now, the most observed coagulopathies in COVID-19 patients are disseminated intravascular coagulation, pulmonary embolism, and deep vein thrombosis which will be discussed in this section individually.

### DIC and PIC

According to current evidence, DIC is not a common condition provided by coronaviruses in patients receiving low molecular weight heparin. however, PIC is suggested to be more important in this patients because cannot be alleviated by heparin.^34^

Lodigiani and colleagues are one of the researcher groups which worked on coagulopathy and observed that only 8 patients out of 388 (2.2%) were detected with DIC.^35^ The low prevalence of overt DIC in COVID-19 patients is also regarded by some other studies.^36-38^

Noteworthy, Tang et al^39^ perused 183 patients and identified that one survivor coupled with 15 non-survivors (out of the total 21) had diagnostic criteria for overt DIC.

In another point of veiw, there are some unclear racial differences which are altering the risk of bing involved with PIC in COVID-19 patients: Caucasian and African-Americans are at a higher risk for having PIC.^34^

Léonard-Lorant and colleagues also reported that 30% of the COVID-19 patients enrolled in their study were diagnosed with acute pulmonary embolism.^40^

### Venous thromboembolism

Besides DIC and PIC, venous thromboembolism (VTE) can also be observed in nearly 25% of COVID-19 patients.^41,42^ Zhang et al^43^ have reported three cases of COVID-19 with augmented partial thromboplastin time, levels of fibrinogen, and d-dimer. As well, they also found IgA, anti–β2-glycoprotein I IgA and IgG in these patients’ blood samples.^43^ Lodgiani et al^35^ also reported that in 7.7% of the patients they examined, Thromboembolic events were detectable. In this study two diagnostic approaches were used: VTE imaging tests and Computed tomography pulmonary angiography.^35^ Although it seems that in ICU patients with SARS-CoV-2 infection, VTE can be more observed (as shown by a study: 47% ^44^). It is proposed that thrombosis prophylaxis should be more considered in ICU patients. ^44^

Another interesting result of their study was the time of thromboembolism diagnosis. “Half of the thromboembolic events were diagnosed within 24 h of hospital admission” They reported.^35^

Both cardiac and respiratory failures are considered as risk factors for VTE ^45^ and thus, it is not still clear that in COVID-19 patients with an underlying cardiac disease VTE occurs because of either of these reasons or both.

### Pulmonary embolism

Investigating died COVID-19 patients shows that one-third of these patients can die directly from pulmonary embolism.^46^ Other studies have also showed a closed number to this statistic.^47,48^

It is shown that D-dimer and c-reactive protein levels are different in COVID patients who develop pulmonary embolism.^49^ An investigation hypothesized “the development of PTE in COVID-19 might be a pulmonary artery thrombosis due to severe lung inflammation and hypercoagulability rather than thromboembolism” for explaining pulmonary embolism in these patients.^49^ In contrast, another study declared that severe manifestations of COVID-19 is more associated with local thrombi in lungs rather than emboli.^50^

### Arterial thromboembolism

Arterial thrombosis is not as common as venous thrombosis^35^ and there are not a great deal of evidence discussing it. However, due to its life-threatening consequences its role in COVID patients’ outcome cannot be neglected. A study on 11 patients detected thrombosis in both small and mid-sized arteries of lungs.^51^ They also expressed that secondary to this event, liver, pancreas, adrenal glands, and kidneys might get involved which highlights the importance of arterial thrombosis in these patients.^51^

Lower limb is another organ in which arterial thrombosis can be observed. Leg ischemia happening due to lower-extremity arterial thrombosis can be a sign of a greater clot burden in patients and therefore, a poorer prognosis.^52^

### Cerebrovascular thrombotic events

In COVID-19, lungs, heart, and kidneys are not the only essential organs which are at the risk of thromboembolic events. Cerebral venous thrombosis (CVT) is another complication which is also providing a poor prognosis in these patients.^53,54^ Multi cerebral vessels seem to be involved by SARS-CoV-2; for instance, straight sinus, deep medullary veins, vein of Galen, and internal cerebral veins are confirmed to get engaged by thrombotic events and hemorrhage.^55^ Ischemic stroke and nonfocal neurological presentations have also been detected in a great percentage of infected people.^53^ The mechanisms of these events are not completely clear, to our knowledge, but it seems that diffuse endothelial inflammation might have a role in this.

### Coaguloathy-related cardiovascular complications in COVID-19

In addition to the issues caused by coagulopathy itself, recent investigations have demonstrated that cardiovascular complications are likely to occur secondary to coagulopathy.

**Cardiac injury.** In this regard, Zhang et al^56^ screened 110 COVID-19 patients and examined different cardiovascular markers in these patients. They identified that there is a relation between cardiac injury in these patients with elevated amounts of D-dimer.^56^ Their study has created a bridge between cardiac injury and coagulopathy in COVID-19 patients. Furthermore, they declared that cardiac injury defined by elevated D-dimer is also increasing the in-hospital mortality of this disease.^56^ Wang et al^57^ showed that in COVID-19 patients if elevated D-dimer and cardiac injury get schronized, worse in-hospital outcomes would be observed.

For more clarifying the mechanisms underlying SARS-CoV-2’s effect on infarction, a case-report study used biopsy on a COVID-19 patient and found that this virus is not only able to localize in lung tissue but also can be load up in other tissues either by viremia or infected macrophages^58^. This mechanism has been approved by other studies, too.^46^ However, this is not the only explanation and vascular-related complications including microthrombi, coronary spasm, and direct vascular injury might also be involved.^59^

### Myocardial infarction

Myocardial infarction (MI) is one of the frequent complications in COVID-19 patients. Investigations show that myocardial infarction shows a relation with ST-segment elevation.^59,60^ It seems that D-dimer levels and troponin are also higher in these patients after infarction.^59^ On the other hand, patients with an underlying ST-segment elevated infarction are in a higher risk of establishing multivessel thrombosis.^60^ It is also possible that chronic impairment of systemic endothelial in patients who already have cardiovascular diseases is worsening the outcome of COVID-19 patients.^61^

### Right ventricular dysfunction

Right ventricular dysfunction (RVD) is another complication of COVID-19 which shows a significant relation to mortality of this disease.^62-64^ A research on 110 COVID-19 patients shows that right ventricular dilation can be detected in 31% of these patients.^62^ Although, Pagnesi et al^65^ did not identify any relation between the severity of COVID-19 and RVD and concluded the opposite.

Considering the mechanisms, generally right ventricular dysfunction or ischaemia can also happen due to pulmonary embolism^66^; however, confirming this statement in COVID-19 patients needs more investigation. As well, Argulian et al^62^ listed these mechanisms for explaining this association: “thrombotic events, hypoxemic vasoconstriction, cytokine milieu, and direct viral damage”.

### Proposed diagnostic approaches

Tang et al^67^ have facilitated the diagnosis of coagulopathy in COVID-19 patients by revealing that D-dimer levels should be measured in these patients instead of platelet count. Although, several studies have tested prothrombin and partial thromboplastin time and were also successful.^36^ They found that non-survivors of COVID-19 have a D-dimer average of 2.12 μg/mL and survivors show a D-dimer average of 0.61 μg/mL.^67^ Another study also approved the association of poor prognosis with high levels of D-dimer in COVID-19 patients.^33^

In this regard, several studies have also detected higher levels of D-dimer in patients with exacerbated COVID-19.^68-70^ However, elevated D-dimer is rare in patients with mild COVID-19.71,72 Conversely, Porfidia and Pola have declared that D-dimer elevation can be the result of some other features of COVID-19 instead of coagulopathy.^73^

### Management strategies

Besides the usage of anticoagulant drugs on COVID-19 patients for determining the efficacy of these drugs, it is also possible to prove the coagulant-inducing property of SARS-CoV-2. Llitjos and colleagues monitored COVID-19 patients who used these drugs from their admission and found that venous thromboembolism is occurring in these patients, as well.^41^ Tang et al^67^ examined heparin on 449 patients with severe COVID-19 and found that heparin can decrease the mortality rate of these patients. However, they found that heparin is not effective on all COVID-19 patients. Indeed, only patients experiencing sepsis-induced coagulopathy or patients who show increased D-dimer have a good response to heparin treatment.^67^ Contrastingly, Yin et al^33^ also treated COVID-19 patients with heparin (low-molecular-weight (LMWH) and unfractionated heparin (UFH)) but did not observe any differences in 28-day mortality due to heparin administration.

Wang et al^74^ tried tissue plasminogen activator on three COVID-19 patients and observed an initial improvement in their partial pressure of oxygen/FiO_2_ (P/F) ratio. Although, this result was temporary and thus, further investigations are needed for the confirmation of this drug’s efficacy. Poggiali et al^75^ used Low-molecular-weight heparin prophylaxis for preventing VTE and were not successful.

## Conclusion

**Figure 1:**
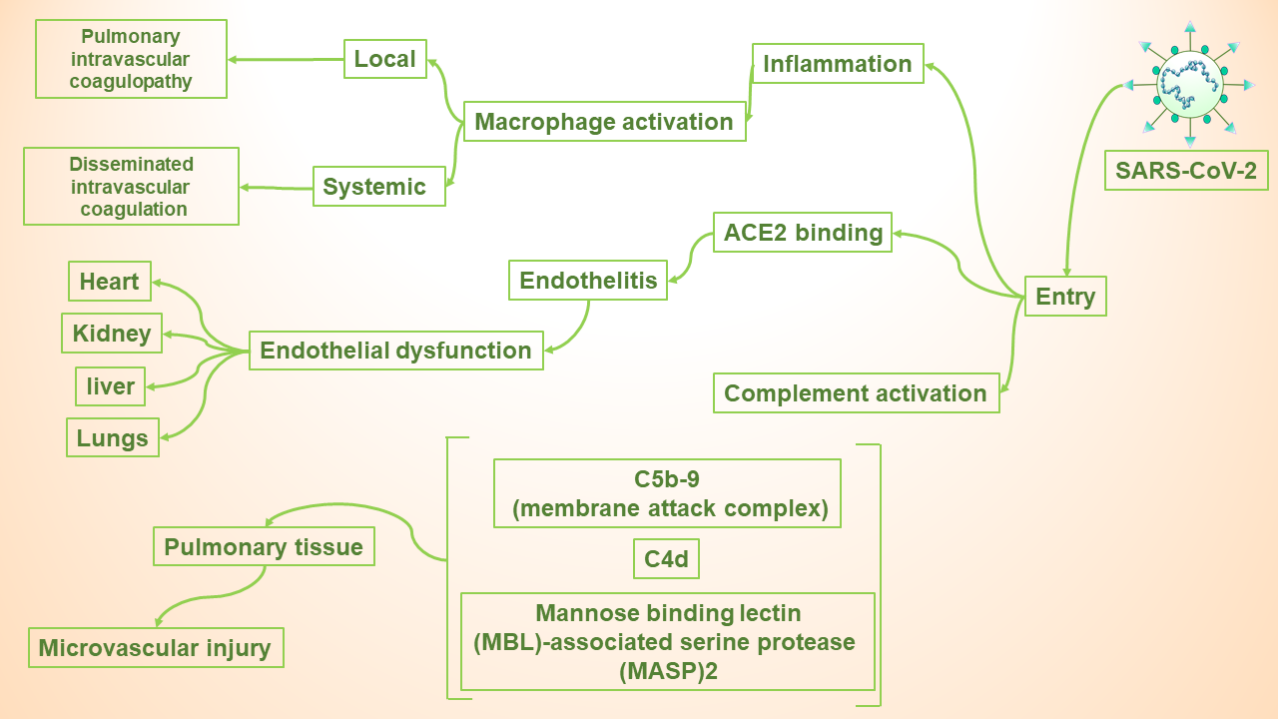
Schematic representation of coronavirus-related diseases pathogenesis, severity, and mortality rate

Since 2002, three outbreaks have happened due to coronavirus infection. Besides the severe acute respiratory syndrome which is their common point, accumulative research has revealed that some other organs or systems of our body can also get involved during coronavirus infection (Figure 1). One of the most lethal post-infection complications is coagulopathy.

## Recommendation

In this review, we have gathered all the evidence on this subject and we suggest the following:

1. A great body of evidence has demonstrated that in patients with exacerbated COVID-19, coagulation tests show an imbalance. This means that coagulopathy plays a pivotal role in disease severity and mortality. On the other hand, this fact might be explainable by induced inflammation and the functions and expression sites of ACE2, a SARS-CoV-2 receptor.
2. Disseminated intravascular coagulation, pulmonary embolism, and deep vein thrombosis are three major coagulopathies occurring in COVID-19 patients due to viral infection.
3. Due to the effect of coagulopathy on COVID-19 mortality rate, we suggest that physicians should have this condition in mind and be more vigilant and monitor COVID patients regularly with coagulation tests. Furthermore, it is approved that D-dimer and fibrinogen levels, as well as prothrombin time, are more effective than platelet count; thus, they would provide an earlier diagnosis. Furthermore, more tests such as compression ultrasound and echocardiography should be used more widely in these patients in both early and late stages.^75^
4. In the treatment point of view, two drugs have been suggested: heparin and tissue plasminogen activator. However, there are antithetical studies about the efficacy of heparin and there are not enough researches using tissue plasminogen activator. Hence, still, more investigations are needed for coming to a definite conclusion about treating COVID-related coagulopathy.

## Competing interests

The authors declare no conflict of interest.

## Funding

None.
